# Screening for iron deficiency and iron deficiency anaemia in pregnancy: a structured review and gap analysis against UK national screening criteria

**DOI:** 10.1186/s12884-015-0679-9

**Published:** 2015-10-20

**Authors:** Ruramayi Rukuni, Marian Knight, Michael F Murphy, David Roberts, Simon J Stanworth

**Affiliations:** National Perinatal Epidemiology Unit, University of Oxford, Old Road, Campus, Oxford, OX3 7LF UK; Department of Haematology, John Radcliffe Hospital, NHS Blood & Transplant/Oxford University Hospital Trust, University of Oxford, Oxford, UK

**Keywords:** Anaemia, iron, iron deficiency, pregnancy, screening

## Abstract

**Background:**

Iron deficiency anaemia is a common problem in pregnancy despite national recommendations and guidelines for treatment. The aim of this study was to appraise the evidence against the UK National Screening Committee (UKNSC) criteria as to whether a national screening programme could reduce the prevalence of iron deficiency anaemia and/or iron deficiency in pregnancy and improve maternal and fetal outcomes.

**Methods:**

Search strategies were developed for the Cochrane library, Medline and Embase to identify evidence relevant to UK National Screening Committee (UKNSC) appraisal criteria which cover the natural history of iron deficiency and iron deficiency anaemia, the tests for screening, clinical management and evidence of cost effectiveness.

**Results:**

Many studies evaluated haematological outcomes of anaemia, but few analysed clinical consequences. Haemoglobin and ferritin appeared the most suitable screening tests, although future options may follow recent advances in understanding iron homeostasis. The clinical consequences of iron deficiency without anaemia are unknown. Oral and intravenous iron are effective in improving haemoglobin and iron parameters. There have been no trials or economic evaluations of a national screening programme for iron deficiency anaemia in pregnancy.

**Conclusions:**

Iron deficiency in pregnancy remains an important problem although effective tests and treatment exist. A national screening programme could be of value for early detection and intervention. However, high quality studies are required to confirm whether this would reduce maternal and infant morbidity and be cost effective.

**Electronic supplementary material:**

The online version of this article (doi:10.1186/s12884-015-0679-9) contains supplementary material, which is available to authorized users.

## Background

Anaemia in pregnancy is a worldwide problem. The incidence and aetiology vary considerably between low and high-income countries, with iron deficiency considered the most common cause in the latter. Somewhat surprisingly, recent prevalence estimates of maternal anaemia in pregnancy in the UK and other high-income countries, suggest there has been no significant decrease in the prevalence of anaemia in pregnancy in the last decade [1]. The UK prevalence of maternal anaemia in the antenatal period was estimated as 24 % in a recent cross sectional study [2].

There is a spectrum of iron deficiency in pregnancy ranging from iron depletion without anaemia (absent iron stores with a normal haemoglobin concentration) to overt anaemia [3]. Although iron deficiency in pregnancy is, in principle, identifiable, treatable and possibly preventable with iron supplementation, there is uncertainty about its significance as a clinical and public health problem, and whether systematic screening and treatment for iron deficiency and iron deficiency anaemia in pregnancy would improve maternal and infant outcomes.

The UK guidelines that underpin the clinical management of iron deficiency anaemia in pregnancy are summarised in Table 1. [4–6]. There are also a variety of international and national guidelines. Guidance from the World Health Organisation [7], United States of America [8, 9], Australia and New Zealand [10] has been reviewed elsewhere [11, 12]. Recommendations for current practice in the UK are to assess the mother’s haemoglobin concentration at booking and at 28 weeks’ gestation and ensure there are systems in place to follow up abnormal results [4]; routine assessment of iron status is not recommended in all women [5].

**Table 1:** Summary of existing guidance for the management of anaemia or iron deficiency anaemia (IDA) in pregnancy in the UK

Body	Year	Title	Recommendations in the antenatal period (evidence level)
British Committee for Standards in Haematology [5]	2012	UK guidelines on the management of iron deficiency in pregnancy	• Women with Hb <110 g/l or <105 g/l in the second and third trimesters, should have a trial of oral iron as the first line diagnostic test for microcytic or normocytic anaemia; an increased Hb after two weeks of therapy is taken to be confirmatory (1B).
			• Refer to secondary care for further investigation for other causes of anaemia if Hb does not improve after 2 weeks, severe <70 g/l, significant symptoms or late gestation (>34 weeks) (2B).
			• Routine ferritin testing in non-anaemic pregnant women is not recommended unless they are ‘at risk’ of iron deficiency (2B).
			• Treatment is suggested where ferritin is <30 mcg/l with rapid review and follow up of results (2A)
			Women at risk include: 1. anaemic women where estimation of iron stores is necessary, e.g. women with haemogloblinopathies or prior to parenteral iron replacement
			2. those who are not anaemic but at risk of iron depletion (previous anaemia, multiparity > 3, consecutive pregnancy, vegetarians, teenage pregnancies, recent history of bleeding)
			3. Non-anaemic women where estimation of iron stores is necessary as significant blood loss may occur (high bleeding risk, Jehovah witnesses).
			• Consider IV iron from the 2^nd^ trimester onwards if absolute non-compliance or intolerance to oral iron or proven malabsorption (1A)
			• All women should receive dietary counselling detailing iron rich foods, inhibitory factors reinforced by provision of an information leaflet (1A)
			• Evidence level AHCPR methodology
National Institute for Health and Care Excellence Clinical [4]	2008	Antenatal Care: routine care for the healthy pregnant woman. Guideline 62.	• Hb should be checked at booking and 28 weeks when other blood screening tests are being carried out (B)
			• nutritional information should be offered to all pregnant women (A)
			• Hb <110 g/l 1^st^ trimester and 105 g/l at 28 weeks should be investigated and iron supplementation considered in indicated (A)
			• iron supplementation should not be offered routinely as there are unpleasant maternal side effects with no clearly demonstrated maternal and infant benefits (A)
			• Evidence level GRADE methodology
Royal College of Obstetricians and Gynaecologists [6]	2007	Blood Transfusions in Obstetrics Green-top 47	• Anaemia should be treated to reduce probability of transfusion requirement (GPP).
			• If Hb <105 g/l in the antenatal period, consider haematinic deficiency (GPP).
			• Once haemoglobinopathies have been excluded, oral iron should be the first-line treatment for iron deficiency (GPP).
			• Parenteral iron is indicated when oral iron is not tolerated, absorbed or patient compliance is in doubt (GPP).
			• Evidence level AHCPR methodology + GPP (clinical good practice point where evidence lacking)

Abbreviations: (*AHCPR*) US Agency for Health Care and Policy Research, (*GRADE*) Grading of Recommendations Assessment, Development and Evaluation; GPP clinical good practice point

Whilst ‘screening’ for iron deficiency anaemia in pregnancy is consistently recommended in these guidelines, the approach in practice differs significantly by country. Screening in this review refers to a systematic integrated programme targeting all pregnant women; including testing, diagnosis, treatment, training and quality assurance. As yet, there has been no formal screening programme to implement these recommendations in the UK.

The aim of this analysis was to review the case for screening pregnant women for iron deficiency and iron deficiency anaemia, using, as a framework, the criteria set out by the UK National Screening Committee [13]. Additional objectives were to identify any gaps in the evidence and outline opportunities for further research to answer the question about the case for screening.

## Methods

The UKNSC screening programme appraisal criteria were accessed through the national UK screening portal [13]. The UKNSC outline 22 criteria that must be met before a systematic population screening programme for a condition is initiated (Additional file 1: Supplement 1). In order to identify relevant literature we searched the Cochrane Library 2014, Medline 1946-August 2014 and Embase 1974 to August 2014. Search strategies were adapted for each database and included combinations of medical subject headings (MeSH terms) relevant to iron deficiency and iron deficiency anaemia in pregnancy combined with free text search terms. Example search strategies are provided (Additional file 2: Supplement 2). Studies not published in English were excluded. Reference lists of relevant studies were screened to identify further literature. The literature was reviewed against the UKNSC criteria and the key gaps in the evidence were identified. Criteria not relevant to iron deficiency and iron deficiency anaemia in pregnancy were excluded, thus criteria 4, 9 and 22 were excluded as they were specific to screening for mutations. Relevant aspects of the PRISMA guidelines were followed when conducting this review.

## Ethics committee approval

Ethical approval was not required for this literature review.

## Results

Gaps in the evidence were identified in relation to a number of criteria as summarised in Table 2. Results for criteria relating to policies for diagnostic investigation and treatment of iron deficiency and iron deficiency anaemia were presented together as they are discussed simultaneously in UK clinical guidelines. Results for the criteria for adequacy of staffing and facilitates and clinical management prior to commencement of a screening programme were also presented together.

**Table 2:** Literature review results for maternal iron deficiency and iron deficiency anaemia against UKNSC Criteria

## The condition

### The condition must be an important health problem

Iron deficiency is the most common nutritional deficiency in the world and represents the most common cause of maternal anaemia [14]. Pregnant women and children are particularly vulnerable to iron deficiency anaemia due to the high demand for iron associated with growth. A number of studies were identified suggesting that there is a high prevalence of iron deficiency anaemia in pregnancy in the UK [2, 15].

Multiple studies of maternal anaemia have evaluated haematological outcomes and/or measures of iron status, however a limited number of these have evaluated clinical consequences. Table 3 summarises a number of important clinical outcomes of iron deficiency anaemia reported in the literature. It also gives an indication of the quality of the evidence based on available systematic review evidence as assessed using GRADE methodology. A key information gap is the lack of clinical outcomes as reported by women themselves.

**Table 3:** Clinical outcomes associated with maternal iron deficiency anaemia

Maternal outcomes		Infant outcomes	
Evidence grade high (4)		Evidence grade high (4)	
Evidence grade moderate (3)		Evidence grade moderate (3)	
Postpartum infection		Low birth weight	
		Preterm delivery (<37wks)	
Evidence grade low (2)		Evidence grade low (2)	
Infection during pregnancy		Mean birth weight	Special care admission
		Very low birth weight	Birth length
		Neurodevelopmental delay	Head circumference at birth
		Neonatal death	Small for gestational age
		Congenital anomaly	Very premature birth (<34wks)
Evidence grade very low (1)		Evidence grade very low (1)	
Antepartum haemorrhage	Placental malaria	Still birth	
Placental abruption	Transfusion		
Postpartum haemorrhage	Premature rupture of membranes		
Breast feeding duration	Pre-eclampsia		
Maternal wellbeing	Reduced cognitive ability		
Maternal death	Post-partum depression		
Maternal malaria	Emotional instability		
Side effects	Lactation failure		

The majority of studies analysing the impacts of iron status tend to comprise populations with iron deficiency anaemia. Iron deficiency *per se* is thought to affect tissue oxidative capacity, whereas iron deficiency anaemia also affects oxygen carrying capacity [16]. Whilst there are placebo controlled trials in high income settings that have shown that iron supplementation is effective at preventing anaemia in pregnancy [3, 17]; there are no published randomised trials that have evaluated the clinical effects of iron deficiency without anaemia on maternal and infant outcomes in pregnancy. Evidence from one small cohort study in a high income country suggested a large proportion of pregnant women with normal haemoglobin have iron depletion, which is associated with postnatal depression [18]. There is randomised trial evidence of the clinical effects of iron deficiency alone from other population groups, which may be relevant in pregnancy e.g. iron deficiency associated with fatigue in menstruating women [19]; with impaired physical performance in female athletes and with reversible cognitive and behavioural deficits in early infant development [20]. Maternal iron deficiency is associated with low neonatal iron stores in the neonate [21] and it has been suggested that the period of particular vulnerability for iron deficiency in the fetus and newborn is between the last trimester and the first two years of life [22].

### The epidemiology and natural history of the condition, including development from latent to declared disease, should be adequately understood and there should be a detectable risk factor, disease marker, latent period or early symptomatic stage

There are limited studies describing the epidemiology of anaemia in pregnancy in the UK. Although haemoglobin in normally measured in pregnancy, these data are not as yet routinely compiled and analysed. Predictors of iron deficiency and iron deficiency anaemia in pregnancy identified from cross-sectional studies include young maternal age, previous pregnancy and ethnicity [2]. Health Survey for England (HSE) data from 2004 also showed the prevalence of anaemia, irrespective of cause, varied between ethnic groups. The prevalence of anaemia was highest in Indian women at 29 %, while it was 16 % in Black Caribbean women and 6-7 % in Irish and Chinese women. Unfortunately the HSE data does not describe iron status in these groups [23]. In some ethnic groups, the high incidence of haemoglobinopathies, particularly alpha- and beta- thalassaemia trait may be associated with lower haemoglobin and contribute the high rates of microcytic anaemia in pregnancy [24].

There is some evidence that specific dietary factors play a role in anaemia. Vegetarians have lower iron stores but show no differences in haemoglobin indices compared to those who eat meat [25]. Other factors identified in the literature that place women from high-income countries in their reproductive years at risk of iron deficiency and iron deficiency anaemia are menstruation and intra-uterine contraceptive devices without hormonal preparations. These devices are thought to contribute to iron deficiency by increasing menstrual blood loss by 30 - 50 % [26]. Other factors include weight, smoking status, blood donation intensity and previous pregnancies [27].

Early iron deficiency is characterised by diminished iron stores. This becomes iron depletion when iron stores are absent. Iron deficiency anaemia represents the late stage of this spectrum. Although mild or moderate iron deficiency and or anaemia may be asymptomatic, there are a variety of blood iron indicators that exist to detect early stages of disease.

### All the cost-effective primary prevention interventions should have been implemented as far as practicable

Relevant primary prevention measures include iron fortification of foods and improved diet. Early studies have evaluated the relative effectiveness and cost per DALY of prevention strategies such as prenatal supplementation, universal supplementation and universal fortification. It is thought that diet based approaches and targeted supplementation is particularly cost-effective and that food fortification achieves the highest benefit-to-cost ratio [7]. There is evidence from systematic reviews that fortification of foods with iron results in a statistically significant improved haemoglobin and iron status in pregnancy [28]. Iron fortification has been implemented in the UK for a variety of foods including flour and cereals and there is a legal minimum iron level in bread and flour [29]. Routine prophylactic iron supplementation in pregnancy has not been recommended in the UK due to concerns about side-effects, poor compliance and potential adverse effects of excess iron [4]. There is evidence from a systematic review that nutrition education counselling improves maternal anaemia [30], but it is unclear how widely such counselling occurs, despite UK national guidelines encouraging improved dietary iron intake during pregnancy.

## The test

### There should be a simple, safe, precise and validated screening test

Table 4 summarises the characteristics of available iron indicators. In clinical practice low haemoglobin and low mean cell volume are widely used to identify iron deficiency anaemia but are not specific and may be found in other conditions such as thalassaemia trait and chronic disease. WHO/CDC technical guidance based on systematic review evidence is that, in the absence of infections, serum ferritin or serum transferrin receptor in combination with haemoglobin provides the best approach to measuring iron status in general populations [31]. However, serum transferrin receptor is not widely available or used in clinical practice in many countries. A formal study of the accuracy of diagnostic tests for iron deficiency and a study to determine the diagnostic accuracy of erythrocyte indices and ferritin at predicting therapeutic responsiveness to oral iron therapy in pregnancy is underway [32]. Recent advances in the understanding of iron metabolism may define new options for screening tests. Hepcidin is a novel peptide hormone, whose level falls in iron deficiency and so allows the uptake of iron from the small intestine. Low hepcidin levels may prove useful test to predict response to absorption of oral iron in iron deficiency [33]. An additional benefit is that, unlike ferritin, low hepcidin levels, indicating iron deficiency, are not increased by inflammation [34].

**Table 4:** Characteristics of available iron status indicators adapted from [31]

Indicator	Commonly available	Complexity	Sampling variability	Validated reference material for pregnancy
Haemoglobin	Y	Low	Low	Available
Reticulocyte haemoglobin content	N	Low	Low	Available
Zinc protoporphyrin	Y	Low	Med	Not available
Mean cell volume	Y	Low	Low	Available
Transferrin receptor	N	Medium	Medium	Not available
Hepcidin	N	Medium/High	Unknown	Not available
Ferritin	Y	Medium	Medium	Available

### The distribution of test values in the target population should be known and suitable cut-off level defined and agreed

The distribution and cut-off levels for haemoglobin and ferritin are well defined within UK guidelines. Although anaemia in pregnancy is defined as haemoglobin <110 g/l at any stage of pregnancy by the WHO [35], UK antenatal guidance [4] and CDC guidance [36] define anaemia as <110 g/l in the first trimester and <105 g/l in the second or third trimester.

A ferritin level of 15 mcg/l defines iron deficiency and level or 12 mcg/l iron depletion [31]. A serum ferritin level of <15 mcg/l has a specificity of 98 % and a sensitivity of 75 % for frank iron deficiency with the ‘gold standard’ being bone marrow aspirate in women of reproductive age [37]. The relationship between these thresholds and clinical effects is uncertain. A variety of treatment levels have been suggested in different studies [3]. The agreed treatment level in UK guidelines is based on evidence that a ferritin below 30 mcg/l indicates early iron depletion that will worsen unless treated. However this is based on RCT evidence in a pregnant population with a high prevalence of endemic infection [38].

### The test should be acceptable to the population

There are currently no studies that have formally evaluated the acceptability of blood testing for iron deficiency and iron deficiency anaemia in pregnancy. However, routine blood testing for haemoglobin is already carried out nationally as part of national antenatal guidelines. Factors found to affect the acceptability of testing from qualitative data from UK feasibility studies of thalassaemia screening in pregnancy include emotional and cognitive impact, anxiety, feelings about the result and concern for the baby’s health [39].

## The treatment

### There should be an agreed policy on the further diagnostic investigation of individuals with a positive test result and on the choices available to those individuals

#### There should be agreed evidence based policies covering which individuals should be offered treatment and the appropriated treatment to be offered

There is guidance from the UK National Institute for Health and Care Excellence [4] and UK Royal College of Obstetricians and Gynaecologists [6] for the management of antenatal anaemia in relation to blood transfusion in pregnancy. Specific UK guidance for the further diagnosis and management of iron deficiency in pregnancy is from the British Committee for Standards in Haematology [5]. Within this guideline, haemoglobin and mean cell volume are the considered as the screening test for iron deficiency in pregnancy. Response to oral iron therapy is considered as diagnostic of iron deficiency anaemia. This is a pragmatic approach. Women who do not have anaemia do not have their iron status routinely assessed. The guidelines suggest ferritin in testing for non-anaemic women defined at risk i.e. those with previous anaemia in pregnancy, multiparity (>3), consecutive pregnancy, vegetarians, teenage pregnancies, recent history of bleeding and finally those where estimation of iron stores is necessary as significant blood loss may occur (high bleeding risk, Jehovah’s witnesses). The guidance suggests there should be systems in place for rapid review and follow up of blood results to facilitate treatment of those with a ferritin of <30 mcg/l.

### There should be an effective treatment or intervention for patients identified through early detection, with evidence of early treatment leading to better outcomes than late treatment

There are a variety of treatment options for iron deficiency and iron deficiency anaemia in early pregnancy. These include oral iron, parenteral iron (intravenous and intramuscular preparations) as shown in Table 5. A review of 27 systematic reviews of treatments for maternal anaemia has summarised key gaps in the literature [40]. These included a lack of trials reporting clinical symptoms and consequences of anaemia and highlighted ongoing uncertainty regarding optimal dosing and regimen for iron supplementation in pregnancy. More recently, a systematic review and meta-analysis of 48 randomised trials and 44 cohort studies [41] has reported that prenatal iron in the context of maternal anaemia increases maternal haemoglobin reduces iron deficiency and reduces low birth weight. These effects showed a linear dose–response relationship at doses of 66mg/day or higher. Only a small number of trials reported effects on other outcomes such as stillbirth, neonatal mortality, gestational diabetes and maternal infections in pregnancy, which precluded meta-analysis. A further systematic review [42] has evaluated the outcomes of intermittent iron therapy on the neurodevelopment of children under 12. Whilst there were demonstrable improvements in haematological indices, studies were too small to demonstrate significant associations between iron supplementation and outcomes such as cognitive development, motor skill development, school performance, physical capacity and height for age. All of the trials were in developing countries.

**Table 5:** Iron treatments available suitable for use in pregnancy

Iron preparation	Year first authorised (UK)	Single/Multiple infusion doses	Safety in pregnancy	Cost
Oral Ferrous sulphate	-	-	1^st^ 2^nd^ 3^rd^ trim	£0.97/28 tablets*^1^
Oral Ferrous fumarate	-	-	1^st^ 2^nd^ 3^rd^ trim	£0.79/28 tablets*^1^
Oral Ferrous gluconate	-	-	1^st^ 2^nd^ 3^rd^ trim	£1.95/20 tablets*^1^
IV iron sucrose (Venofer ®)	1998	Multiple	2^nd^ 3^rd^ trim	£148.48*^2^
IV iron dextran (Cosmofer®)	2001	Single	2^nd^ 3^rd^ trim	£115.57*^2^
IV iron ferric carboxymaltose (Ferinject®)	2007	Single	2^nd^ 3^rd^ trim	£286.5*^2^
IV Ferric iron isomastoside (Monofer®)	2010	Single	2^nd^ 3^rd^ trim	£254.25*^12^

This table does not include over the counter multinutrient supplements that contain iron

*^1^cost of tablets as per British National Formulary 2014 [53]

*^2^cost of cumulative dose as per British National Formulary 2014 [53]. Dose calculated based on assumed patient Hb, patient weight of 65kg and target Hb of 150 g/l and target iron depot store targets as per electronic medicines compendium [54]. Cost does not include diluent or nursing time

A Cochrane review of treatments for iron deficiency anaemia in pregnancy [43] included 23 trials using different combinations of intravenous, oral and intramuscular iron. Oral iron therapy was associated with higher rates of withdrawal from studies due to side effects and associated poor compliance. Intravenous iron led to greater improvements in haematological indices, fewer problems with gastrointestinal side effects and better compliance; the trials did not assess clinical consequences. However, it should be noted that IV iron use is only recommended in the second trimester for safety reasons.

There is some evidence largely from cohort studies of a U-shaped association between haemoglobin levels and poor outcomes. Low birth weight, preterm delivery and neonatal death were lowest at haemoglobin <90 g/L and increased over 130 g/L [44–46].

### Adequate staffing and facilities for testing, diagnosis, treatment and programme management should be available prior to the commencement of the screening programme

#### Clinical management of the condition and patient outcomes should be optimised in all health care providers prior to participation in a screening programme

There is limited published evidence suggesting that management of maternal iron deficiency and iron deficiency anaemia in the UK is not optimal. A cross-sectional, multicentre study found that of women found to be anaemic during antenatal visits, only 32 % had their ferritin level checked. Where the ferritin level was found to be <30 mcg/l, only half of women had iron prescribed (Barroso, et al. 2011). There was wide variation in the implementation of existing guidance between the centres. Although barriers to anaemia control strategies have been described in the literature for low and middle income countries [47], these are not well described for high income countries.

## The screening programme

### There should be evidence from high quality randomised controlled trials that the screening programme is effective in reducing mortality or morbidity

There have been no screening programmes or randomised trials of screening programmes for iron deficiency or iron deficiency anaemia in pregnancy in the published literature. There are some studies that have evaluated screening for iron deficiency anaemia in infants [48, 49] and male and female adolescents in the USA [50] with little evidence of benefit.

### There should be evidence that the complete screening programme (test, diagnostic procedures, treatment or intervention is clinically, socially and ethically acceptable to health professionals and the public

There have been no trials that have evaluated the clinical, social or ethical acceptability of a screening programme to treat iron deficiency and iron deficiency anaemia in pregnancy. Factors identified in the literature that would affect the clinically acceptability have been discussed with respect to evidence for clinical outcomes in section 2 and 7. There is reason to believe that an effective programme would be considered socially and ethically acceptable from experience with the thalassaemia and sickle screening antenatal programmes in the UK [39].

### The benefit from the screening programme should outweigh the physical and psychological harm (caused by the test, diagnostic procedures and treatment)

The anticipated benefits of such a programme would include the early identification of latent iron deficiency or iron deficiency anaemia, and would lead to improved maternal and infant outcomes. Potential causes of harm related to screening include false positive results and associated anxiety. False positive diagnoses of anaemia and or iron deficiency may lead to inappropriate iron supplementation. Although it might be anticipated that the benefits would outweigh the risks, we did not identify any formal evaluations of screening programmes for maternal iron deficiency and/or anaemia.

### The opportunity cost of the screening programme (including testing, diagnosis and treatment, administration, training and quality assurance) should be economically balanced in relation to expenditure on medical care as a whole (i.e. Value for money). Assessment against these criteria should have regard to evidence from cost benefit and/or cost effectiveness analyses and have regard to the effective use of available resource

There have been no economic studies evaluating the effectiveness of systematic population screening or whole programmes for treating iron deficiency or iron deficiency anaemia in pregnancy. It is difficult to evaluate the economic implications in the absence of unequivocal evidence of benefit on clinical outcomes in pregnancy.

### All other options for managing the condition should have been considered (e.g. improving treatment, providing other services), to ensure that no more cost-effective intervention could be introduced or current interventions increased within the resources available

Primary prevention of iron deficiency and iron deficiency anaemia in the UK has been described in section 3. Routine supplementation with oral iron tablets in the UK not recommended as it is in other countries such as the US. There are no further interventions known to be more cost effective that could be introduced in the UK.

### There should be a plan for managing and monitoring the screening programme and an agreed set of quality assurance standards. Adequate staffing and facilities for testing, diagnosis, treatment and programme management should be available prior to the commencement of the screening programme

Whilst there have been no randomised trials of a screening programme, other similar antenatal screening programmes e.g. sickle cell and thalassaemia, have comprehensive plans for monitoring and agreed quality assurance standards [51]. There have been no formal studies to establish adequate staffing testing, diagnosis and treatment for a programme that would address iron deficiency and iron deficiency anaemia.

### Evidence-based information, explaining the consequences of testing, investigation and treatment, should be made available to potential participants to assist them in making an informed choice

There is no evidence-based information specific to a screening programme for iron deficiency or iron deficiency anaemia in pregnancy. The infrastructure for delivering information to participants to assist them in making an informed choice exists within antenatal and postnatal care in the UK National Health Service (NHS). The characteristics of what makes information accessible and acceptable to pregnant women in allowing them to make an informed choice has been studied in the context of antenatal screening programmes for other conditions such as thalassaemia [39].

### Public pressure for widening the eligibility criteria, for reducing the screening interval and for increasing the sensitivity of the testing process, should be anticipated. Decisions about these parameters should be scientifically justifiable to the public

The sensitivity of the testing process may be increased as ferritin testing in pregnancy becomes more widespread in practice. The sensitivity of the testing process may also be improved, if and when, newer iron status indicators such as hepcidin become more affordable and widely available. Given the side effects of oral iron, it is possible that the eligibility criteria for those requiring IV iron may widen.

## Discussion

This review has identified a number of significant gaps in the evidence base required to evaluate the case for a structured or coherent national screening programme for iron deficiency or iron deficiency anaemia in maternity in the UK. This is a common problem: despite national guidelines, studies continue to document a high burden of anaemia in pregnancy; and a recent multicentre study in UK documented an overall prevalence of maternal anaemia at 24 % during the antenatal period [2]. There is evidence that poor outcomes such as low birth weight and post-partum infection are associated with iron deficiency anaemia. However, evidence for the association of iron deficiency anaemia or iron deficiency with other important clinical outcomes, including maternal well-being, are lacking or inconsistent, While there is good evidence of improvements in haematological indices with iron supplementation, it is not clear what size of effect early detection and iron supplementation would have on improved clinical outcomes. Oral and intravenous iron are associated with side effects but the optimal strategy for effective dosing and administration, to maximize compliance, are not known. The natural history and effects of iron depletion (without anaemia) have not been defined and there have been no robust evaluations of the cost-effectiveness of screening programmes for iron deficiency anaemia.

## Strengths and limitations

The main strength of this study is that it provides a comprehensive analysis of the gaps in the scientific literature with particular reference to the UK against the UKNSC screening criteria and so highlights key policy and research areas that need to be addressed to reduce the prevalence of iron deficiency and iron deficiency anaemia in pregnancy. A full systematic review on all elements of the National Screening Committee criteria was beyond the scope of this article; we carried out a structured review to highlight the key evidence addressing each criterion. An additional limitation was the exclusion of papers that were not published in the English language.

## Research recommendations

Priorities for research should be directed towards the evidence gaps highlighted in Table 2. These include: changes in the biology of systemic iron homeostasis during pregnancy and the natural history of iron depletion, prognostic evaluations of different diagnostic markers of iron status such as ferritin, soluble transferrin receptor, zinc protoporphyrin and hepcidin which change during pregnancy, the clinical consequences of iron depletion or anaemia for the mother and infant. However, a crucial gap exists between recommendations from clinical guidelines and clinical practice indicating the need for further research to understand the barriers to implementation of practice guidelines in maternity. A better understanding of the issues women feel relevant to their care is important and likely to have implications for compliance with therapy. Future studies should include patient reported outcome measures and functional or quality of life measures relevant to anaemia during pregnancy and the post-partum period.

The major gaps in the evidence for a screening programme for iron deficiency and iron deficiency anaemia are of data from high quality randomised controlled trials that the screening programme is effective in reducing mortality or morbidity, that the benefit from the screening programme should outweigh the physical and psychological harm (caused by the test, diagnostic procedures and treatment) and that the opportunity cost of the screening programme should be economically balanced in relation to expenditure on medical care as a whole (i.e. Value for money).

It is clear, that randomised trials comparing different policies for anaemia recognition and treatment are required; including evaluation of a more robust screening programme against conventional best practice which would provide high quality evidence as to whether better clinical outcomes would be achieved with screening. An alternative approach would be to consider whether targeted screening would be beneficial in women considered to be at high risk of iron deficiency in pregnancy. An initial pilot study could be designed to assess whether screening with iron replacement would lead to improved clinical outcomes. These studies should be coupled with economic analyses to evaluate the opportunity costs and whether the clinical benefits would offset the costs of implementing and monitoring such a programme. Within the UK, there is the opportunity to design studies that evaluate some of these questions in relation to the existing national antenatal haemoglobinopathy screening programme.

## Conclusion

Iron deficiency and iron deficiency anaemia are common in pregnancy yet the clinical consequences; particularly for mild and moderate anaemia, remain poorly understood. Concerted efforts are required to evaluate the case for screening for iron deficiency and iron deficiency anaemia in pregnancy.

## Additional files

Additional file 1: Supplement 1. UK National Screening Committee Criteria for appraising the viability, effectiveness and appropriateness of a screening programme. (DOCX 17 kb) Additional file 2: Supplement 2: Example Search Strategies. (DOCX 21 kb)

## Abbreviations

CDC: Centres for Disease Control and Prevention
FBC: Full blood count
Hb: Haemoglobin
HSE: Health Survey for England
ID: Iron deficiency
IDA: Iron deficiency anaemia
NICE: National Institute for Health and Care Excellence
NHS: National Health Service
UKNSC: UK National Screening Committee
WHO: World Health Organisation
