# Supplementary material for: Screening for iron deficiency and iron deficiency anaemia in pregnancy: a structured review and gap analysis against UK national screening criteria
Source: BMC Pregnancy Childbirth. 2015 Oct 20;15:269. doi: 10.1186/s12884-015-0679-9 (PMC4618150; doi:10.1186/s12884-015-0679-9)
Supplement: Additional file 1: Supplement 1. — UK National Screening Committee Criteria for appraising the viability, effectiveness and appropriateness of a screening programme. (DOCX 17 kb) [file 12884_2015_679_MOESM1_ESM.docx]

**SUPPLEMENT 1**

**UK National Screening Committee Criteria for appraising the viability, effectiveness and appropriateness of a screening programme**

The Condition

1. The condition should be an important health problem

2. The epidemiology and natural history of the condition, including development from latent to declared disease, should be adequately understood and there should be a detectable risk factor, disease marker, latent period or early symptomatic stage.

3. All the cost-effective primary prevention interventions should have been implemented as far as practicable

4. If the carriers of a mutation are identified as a result of screening the natural history of people with this status should be understood, including the psychological implications.

The Test

5. There should be a simple, safe, precise and validated screening test.

6. The distribution of test values in the target population should be known and a suitable cut-off level defined and agreed.

7. The test should be acceptable to the population.

8. There should be an agreed policy on the further diagnostic investigation of individuals with a positive test result and on the choices available to those individuals.

9. If the test is for mutations the criteria used to select the subset of mutations to be covered by screening, if all possible mutations are not being tested, should be clearly set out.

The Treatment

10. There should be an effective treatment or intervention for patients identified through early detection, with evidence of early treatment leading to better outcomes than late treatment.

11 There should be agreed evidence based policies covering which individuals should be offered treatment and the appropriate treatment to be offered.

12. Clinical management of the condition and patient outcomes should be optimised in all health care providers prior to participation in a screening programme.

The Screening Programme

13. There should be evidence from high quality Randomised Controlled Trials that the screening programme is effective in reducing mortality or morbidity. Where screening is aimed solely at providing information to allow the person being screened to make an “informed choice” (e.g. Down’s syndrome, cystic fibrosis carrier screening), there must be evidence from high quality trials that the test accurately measures risk. The information that is provided about the test and its outcome must be of value and readily understood by the individual being screened.

14. There should be evidence that the complete screening programme (test, diagnostic

procedures, treatment/ intervention) is clinically, socially and ethically acceptable to health professionals and the public.

15. The benefit from the screening programme should outweigh the physical and psychological harm (caused by the test, diagnostic procedures and treatment).

16. The opportunity cost of the screening programme (including testing, diagnosis and treatment, administration, training and quality assurance) should be economically balanced in relation to expenditure on medical care as a whole (i.e. value for money). Assessment against these criteria should have regard to evidence from cost benefit and/or cost effectiveness analyses and have regard to the effective use of available resource.

17. All other options for managing the condition should have been considered (e.g. improving treatment, providing other services), to ensure that no more cost effective intervention could be introduced or current interventions increased within the resources available.

18. There should be a plan for managing and monitoring the screening programme and an agreed set of quality assurance standards.

19. Adequate staffing and facilities for testing, diagnosis, treatment and programme management should be available prior to the commencement of the screening programme.

20. Evidence-based information, explaining the consequences of testing, investigation and treatment, should be made available to potential participants to assist them in making an informed choice.

21. Public pressure for widening the eligibility criteria, for reducing the screening interval, and for increasing the sensitivity of the testing process, should be anticipated. Decisions about these parameters should be scientifically justifiable to the public.

22. If screening is for a mutation the programme should be acceptable to people identified as carriers and to other family members.
