# Supplementary material for: Screening for iron deficiency and iron deficiency anaemia in pregnancy: a structured review and gap analysis against UK national screening criteria
Source: BMC Pregnancy Childbirth. 2015 Oct 20;15:269. doi: 10.1186/s12884-015-0679-9 (PMC4618150; doi:10.1186/s12884-015-0679-9)
Supplement: Additional file 2: Supplement 2: — Example Search Strategies. (DOCX 21 kb) [file 12884_2015_679_MOESM2_ESM.docx]

**SUPPLEMENT 2: Literature Search Strategies**

**SCOPING REIVEW LITERATURE SEARCH STRATEGIES**

**COCHRANE DATABASE**

#1 MeSH descriptor: [Anemia, Iron-Deficiency] explode all trees

#2 MeSH descriptor: [Ferritins] explode all trees

#3 MeSH descriptor: [Protoporphyrins] explode all trees

#4 MeSH descriptor: [Iron-Binding Proteins] explode all trees

#5 MeSH descriptor: [Receptors, Transferrin] explode all trees

#6 MeSH descriptor: [Reticulocytes] explode all trees

#7 MeSH descriptor: [Bone Marrow] explode all trees

#8 MeSH descriptor: [Iron] explode all trees

#9 1 or 2 or 3 or 4 or 5 or 6 or 7 or 8

#10 MeSH descriptor: [Anemia] explode all trees

#11 low haemoglobin or low hemoglobin or low red cell count

#12 10 or 11

#13 9 and 12

#14 MeSH descriptor: [Pregnancy] explode all trees

#15 MeSH descriptor: [Maternal Welfare] explode all trees

#16 MeSH descriptor: [Maternal-Child Health Centers] explode all trees

#17 MeSH descriptor: [Pregnancy Complications] explode all trees

#18 MeSH descriptor: [Maternal Health Services] explode all trees

#19 MeSH descriptor: [Midwifery] explode all trees

#20 antenatal or prenatal or perinatal or postnatal or post partum or conception

#21 MeSH descriptor: [Infant] explode all trees

#22 MeSH descriptor: [Child Development] explode all trees

#23 14 or 15 or 26 or 17 or 18 or 19 or 20 or 21 or 22

#24 13 and 23

#25 MeSH descriptor: [Mass Screening] explode all trees

#26 24 and 25

**MEDLINE DATABASE**

1. exp Iron/ or exp Iron Compounds/

2. Ferritins/ad, ae, df, du, de, tu [Administration & Dosage, Adverse Effects, Deficiency, Diagnostic Use, Drug Effects, Therapeutic Use]

3. Folic Acid/ad, ae, du, de, tu, th, to [Administration & Dosage, Adverse Effects, Diagnostic Use, Drug Effects, Therapeutic Use, Therapy, Toxicity]

4. (iron adj2 deficien*).mp. [mp=title, abstract, original title, name of substance word, subject heading word, keyword heading word, protocol supplementary concept, rare disease supplementary concept, unique identifier]

5. (iron adj2 deplet*).mp. [mp=title, abstract, original title, name of substance word, subject heading word, keyword heading word, protocol supplementary concept, rare disease supplementary concept, unique identifier]

6. Anemia, Iron-Deficiency/

7. exp Anemia/

8. (anaemi* or anemi*).mp. [mp=title, abstract, original title, name of substance word, subject heading word, keyword heading word, protocol supplementary concept, rare disease supplementary concept, unique identifier]

9. 1 or 2 or 3 or 4 or 5

10. 7 or 8

11. exp Pregnancy/

12. exp Pregnancy Complications/

13. Maternal Welfare/

14. exp Maternal-Child Nursing/

15. (matern*or prenan* or antenatal or prenatal or perinatal or neoatal).mp. [mp=title, abstract, original title, name of substance word, subject heading word, keyword heading word, protocol supplementary concept, rare disease supplementary concept, unique identifier]

16. Mass Screening/

17. 11 or 12 or 13 or 14 or 15

18. 9 and 10

19. 6 or 18

20. 16 and 17 and 19

**EMBASE DATABASE**

1 exp iron dextran/ or exp iron regulatory protein 1/ or exp iron chelation/ or exp iron restriction/ or exp iron chelate/ or exp iron/ or exp iron saccharate/ or exp iron kinetics/ or exp iron metabolism/ or exp iron therapy/ or exp iron regulatory protein 2/ or exp iron depletion/ or exp iron chelating agent/ or exp iron absorption/ or exp iron binding capacity/ or exp iron deficiency anemia/ or exp iron protein succinylate/ or exp iron intake/ or exp iron balance/ or exp iron metabolism disorder/ or exp iron storage/ or exp iron overload/ or exp iron deficiency/ or exp iron regulatory factor/ or exp iron binding protein/ or exp nonheme iron protein/ or exp iron urine l! evel/ or exp iron transport/ or exp iron salt/ or exp iron complex/ or exp iron derivative/ or exp iron blood level

2 exp anemia/ or exp folic acid/ or exp iron

3 exp iron intake/ or exp iron overload/ or exp iron/ or exp ferritin/ or exp blood/ or exp iron deficiency anemia/

4 exp ferrous sulfide/ or exp ferrous fumarate/ or exp ferrous sulfate/ or exp ferrous chloride/ or exp ferrous ion/ or exp ferrous gluconate/ or exp ferrous sulfate plus multivitamin/

5 exp protoporphyrin zinc/

6 exp transferrin/ or exp transferrin receptor/

8 exp reticulocyte/

9 anaemia.mp. or exp anemia/

10 exp blood cell count/ or low blood count.mp.

11 haematocrit.mp. or exp hematocrit/

12 1 or 2 or 3 or 4 or 5 or 6 or 7 or 8 or 9 or 10 or 11

13 exp pregnancy diabetes mellitus/ or exp time to pregnancy/ or exp pregnancy/ or exp attitude to pregnancy/ or exp ectopic pregnancy/ or exp twin pregnancy/ or exp early pregnancy factor/ or exp prolonged pregnancy/ or exp adolescent pregnancy/ or exp pregnancy outcome/ or exp unplanned pregnancy/ or exp "parameters concerning the fetus, newborn and pregnancy"/ or exp first tri! mester pregnancy/ or exp pregnancy disorder/ or exp third trimester pregnancy/ or exp pregnancy rate/ or exp high risk pregnancy/ or exp unwanted pregnancy/ or exp pregnancy complication/ or exp second trimester pregnancy/ or exp multiple pregnancy/

14 exp maternal diabetes mellitus/ or exp maternal nutrition/ or exp maternal welfare/ or exp maternal morbidity/ or exp maternal mortality/ or exp maternal attitude/ or exp maternal stress/

15 exp prenatal care/ or antenatal.mp.

16 exp perinatal mortality/ or exp perinatal development/ or exp perinatal period/ or exp perinatal death/ or perinatal.mp. or exp perinatal infection/ or exp perinatal asphyxia/ or exp perinatal morbidity/

17 exp postnatal development/ or exp perinatal period/ or post na tal.mp. or exp development/

18 exp puerperal psychosis/ or exp puerperium/ or exp postpartum hemorrhage/ or exp puerperal depression/ or postpartum.mp. or exp puerperal disorder/

19 exp midwife/

20 exp conception/

21 preconception.mp.

22 infant/

23 newborn/

24 child/

25 neonatal.mp.

26 exp child deve! lopment/

27 13 or 14 or 15 or 16 or 17 or 18 or 19 or 20 or 21 or 22 or 23 or 24 or 25 or 26

28 12 and 27

29 exp screening

30 28 and 29

1. limit 30 to english language

**Search Strategy and Results for Cost Analysis Dietary Supplementation:**

Ovid MEDLINE(R) In-Process & Other Non-Indexed Citations and Ovid MEDLINE(R) <1946 to Present>

| # | Search History | Results |
| --- | --- | --- |
| 1 | Anemia, Iron-Deficiency/ | 2311 |
| 2 | Pregnancy/ | 111432 |
| 3 | Dietary Supplements/ or Administration, Oral/ or oral supplementation.mp. | 39223 |
| 4 | 1 and 2 and 3 | 122 |
| 5 | cost.mp. or "Costs and Cost Analysis"/ | 110447 |
| 6 | 4 and 5 | 4 |

**Search Strategy and Results for Reference Values:**

Ovid MEDLINE(R) In-Process & Other Non-Indexed Citations and Ovid MEDLINE(R) <1946 to Present>

| # | Search History | Results |
| --- | --- | --- |
| 1 | Iron/ or Anemia, Iron-Deficiency/ | 17446 |
| 2 | Pregnancy/ | 111432 |
| 3 | 1 and 2 | 655 |
| 4 | (haemoglobin or hemoglobin or ferritin).mp. [mp=title, abstract, original title, name of substance word, subject heading word, keyword heading word, protocol supplementary concept, rare disease supplementary concept, unique identifier] | 40218 |
| 5 | Reference Values/ | 22362 |
| 6 | 4 and 5 | 620 |
| 7 | 3 and 6 | 10 |
